# SurvBal: compositional microbiome balances for survival outcomes

**DOI:** 10.1093/bioinformatics/btae612

**Published:** 2024-10-15

**Authors:** Ying Li, Teresa Lee, Kai Marin, Xing Hua, Sujatha Srinivasan, David N Fredricks, John R Lee, Wodan Ling

**Affiliations:** Division of Biostatistics, Department of Population Health Sciences, Weill Cornell Medicine, New York, NY 10065, United States; Public Health Sciences Division, Fred Hutchinson Cancer Center, Seattle, WA 98109, United States; Public Health Sciences Division, Fred Hutchinson Cancer Center, Seattle, WA 98109, United States; Public Health Sciences Division, Fred Hutchinson Cancer Center, Seattle, WA 98109, United States; Vaccine and Infectious Disease Division, Fred Hutchinson Cancer Center, Seattle, WA 98109, United States; Vaccine and Infectious Disease Division, Fred Hutchinson Cancer Center, Seattle, WA 98109, United States; Department of Medicine, University of Washington, Seattle, WA 98195, United States; Division of Nephrology and Hypertension, Department of Medicine, Weill Cornell Medicine, New York, NY 10065, United States; Department of Transplantation Medicine, New York Presbyterian Hospital–Weill Cornell Medical Center, New York, NY 10065, United States; Division of Biostatistics, Department of Population Health Sciences, Weill Cornell Medicine, New York, NY 10065, United States

## Abstract

**Summary:**

Identification of balances of bacterial taxa in relation to continuous and dichotomous outcomes is an increasingly frequent analytic objective in microbiome profiling experiments. SurvBal enables the selection of balances in relation to censored survival or time-to-event outcomes which are of considerable interest in many biomedical studies. The most commonly used survival models—the Cox proportional hazards and parametric survival models are included in the package, which are used in combination with step-wise selection procedures to identify the optimal associated balance of microbiome, i.e. the ratio of the geometric means of two groups of taxa’s relative abundances.

**Availability and implementation:**

The SurvBal R package and Shiny app can be accessed at https://github.com/yinglia/SurvBal and https://yinglistats.shinyapps.io/shinyapp-survbal/.

## 1 Introduction

Compositional balances have served as a powerful strategy for implicating bacterial taxa in relation to a wide range of outcomes. The underlying principle of the global balances is that outcomes depend on the ratio of two sets of bacterial relative abundances. Thus, selbal (Rivera-Pinto *et al.* 2018) chooses to identify the (log) ratio of the geometric means of two sets of taxa. Essentially, the numerator of the ratio is the geometric mean of the taxa positively correlated with the outcome while the denominator is the geometric mean of the taxa negatively correlated with the outcome. Philosophically, the global balances include better characterization of the idea of dysbiosis as well as better accommodation of the issue of compositionality which creates significant challenges for interpretation. Operationally, the optimal balances can be identified through greedy search procedures. The approach has been successfully used to identify balances, and the comprising taxa, related to many different outcomes including COVID severity (Trøseid *et al.* 2023), neutrophil levels in HIV infection (Hensley-McBain *et al.* 2019), among others.

However, a limitation in the field is the lack of software for identifying balances in relation to censored survival or time-to-event outcomes. Yet, such outcomes are of considerable interest, particularly as human microbiome studies interface with clinical studies in which time-to-event outcomes (e.g. overall survival (OS), time to relapse, time to disease onset, etc.) are the most commonly investigated outcomes. Such studies are often subject to significant censoring such that specialized survival models are needed.

We present SurvBal, a flexible R package that facilitates the analysis of balances with censored time-to-event outcomes. It identifies the log-ratio of the geometric means of two sets of taxa that is most associated with the survival outcome using a greedy step-wise selection approach. The software supports the Cox proportional hazards model and parametric survival models, and by extension, accelerated failure time (AFT) models, and reports a selected global balance of bacteria increasing vs. decreasing the hazard or survival time. A comprehensive Shiny app is provided for broader users to interactively explore the analytical tool.

## 2 Software description

Suppose $X_{i}=(X_{i1},X_{i2},\ldots ,X_{iK})$ is the microbial composition for subject *i*, where $X_{ij}$’s are relative abundances and the sum over the *K* taxa is 1. If we have a subset of bacteria that increase hazard, which is denoted by $X_{+}$, indexed by $I_{+}$ and composed of $k_{+}$ taxa, and another subset decreasing hazard, denoted by $X_{-}$, indexed by $I_{-}$ and composed of $k_{-}$ taxa, the balance is defined as the normalized log ratio of the geometric means of the two groups,

$$B_{i}^{\text{PH}}(X_{+},X_{-})=\sqrt{\frac{k_{+}k_{-}}{k_{+}+k_{-}}}\;\text{log}\;\frac{{\left(\prod_{j\in I_{+}}X_{ij}\right)}^{1/k_{+}}}{{\left(\prod_{j\in I_{-}}X_{ij}\right)}^{1/k_{-}}}.$$

Equivalently, we can have

(1) $$B_{i}^{\text{PH}}(X_{+},X_{-})\propto \frac{1}{k_{+}}\sum_{j\in I_{+}}\;\text{log}\;X_{ij}-\frac{1}{k_{-}}\sum_{j\in I_{-}}\;\text{log}\;X_{ij}.$$

The log-contrast in (1) handles compositionality (Aitchison 1982), accommodating the relative nature of microbiome abundances. Moreover, (1) is a special log-contrast, where the log differences are not included in a linear model additively, but are combined together as a single variable or feature of the microbiome (Rivera-Pinto *et al.* 2018). Via the Cox proportional hazards model, the balance can be associated with the hazard at time *t* as

(2) $$h(t|X_{i},Z_{i})=h_{0}(t)\;\text{exp}\;\{\gamma B_{i}^{\text{PH}}(X_{+},X_{-})+Z_{i}^{⊤}\beta \},$$

where $h_{0}(t)$ is the baseline hazard, γ is the effect size of the balance, $Z_{i}$ denotes the biomedical or demographic covariates to adjust for in the analysis and β is the corresponding effect size. Similarly, if we have a subset of bacteria that increase survival time, $X_{+}^{{}^{\prime }}$, and another subset decreasing survival time, $X_{-}^{{}^{\prime }}$, via a parametric survival model such as the AFT model, the balance can be associated with the survival time $T_{i}$ as

(3) $$\text{log}\;T_{i}=\gamma^{{}^{\prime }}B_{i}^{\text{Parametric}}(X_{+}^{{}^{\prime }},X_{-}^{{}^{\prime }})+Z_{i}^{⊤}\beta^{{}^{\prime }}+\epsilon_{i},$$

where, e.g. $\epsilon_{i}$ could follow the extreme value distribution, i.e. $T_{i}$ follows the Weibull distribution.

**Figure 1.**
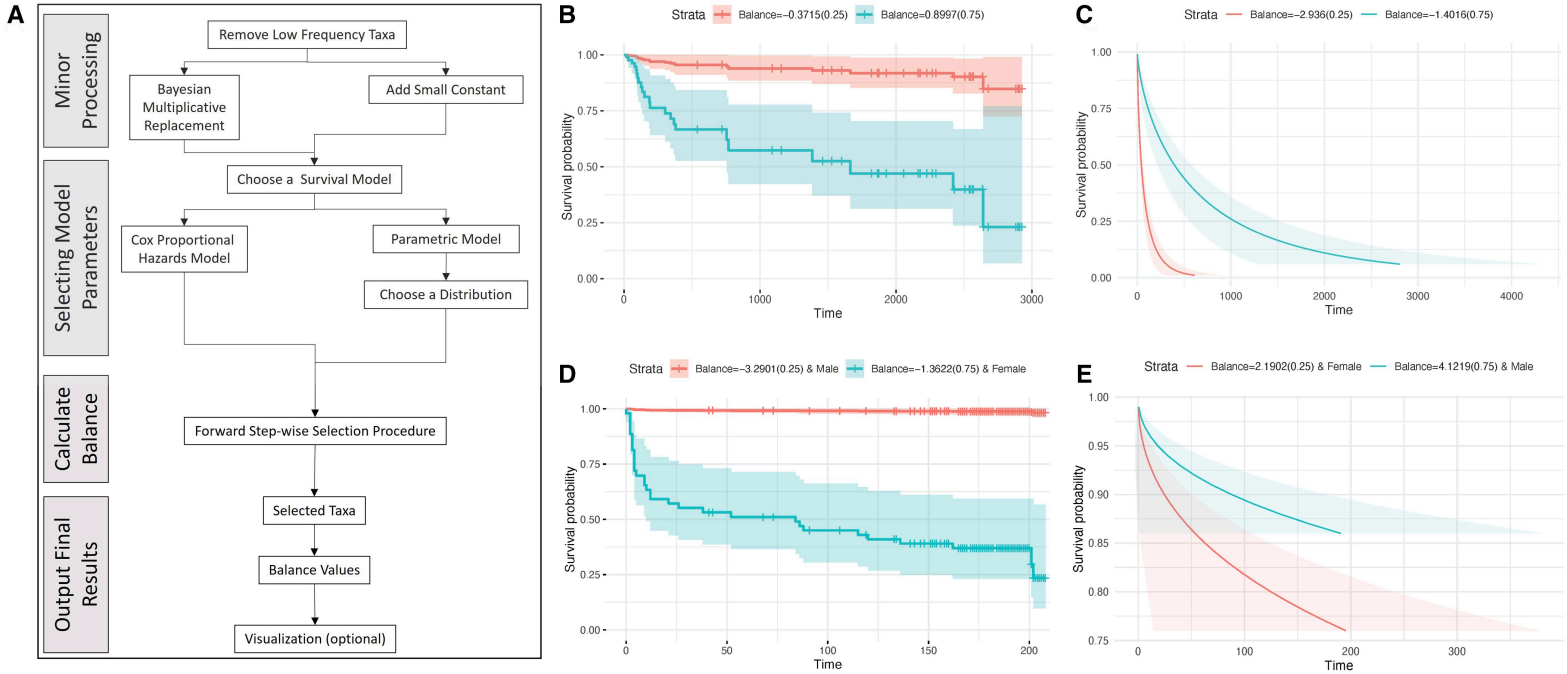
(**A**) Overview of SurvBal. Stratified time-to-event plots by a lower and a higher values of the selected balances of gut microbiome: HCT recipients, (**B**) OS under the Cox model, (**C**) time-to-GvHD under the parametric survival model; kidney transplant recipients, (**D**) time-to-*E.coli* bacteriuria under the Cox model, and (**E**) time-to-*Enterococcus* bacteriuria under the parametric survival model.

SurvBal is a variable selection software designed to identify the two sets of taxa, $X_{+}$ and $X_{-}$ (or $X_{+}^{{}^{\prime }}$ and $X_{-}^{{}^{\prime }}$), that compose the optimal balance of microbiome associated with the interested survival outcome. It takes a matrix consisting of the raw counts of the taxa for each subject in the study, a survival object from the R package “survival” (Therneau and Lumley 2015) that contains survival times and censoring indicators, and, if applicable, the covariates to adjust for. The software is flexible as additional options are available throughout the model building and variable selection processes—from the pre-processing to the final selection of the microbial balance. Figure 1A provides an overview of SurvBal with the details deferred to Section 1 of the Supplementary Information. We carried out extensive simulation studies (Section 2 of the Supplementary Information) to demonstrate the reliability of SurvBal (measured by precision and recall) in selecting the balance of taxa for survival outcomes.

The greedy algorithm of SurvBal may still select a balance even when no associations exist, while real-world microbiome studies frequently show no link between the microbiome and survival outcomes. To address this, SurvBal performs a global community-level association test, MiRKAT-S (Plantinga *et al.* 2017), before selecting the microbial balance. Specifically, we encode microbiome data in ecologically informative distance metrics (Bray-Curtis and Jaccard distances), then use MiRKAT-S to compare the similarity in microbiota to the similarity in survival times between subjects. The test reports an omnibus *P*-value, indicating whether there is a significant association between the survival outcome and the presence-absence status or the abundance of the microbial profile. It is worth noting that the community-level testing identifies global shifts in the microbial profile and is best used when there are concerted differences among a large number of taxa. On the other hand, analysis of balances focuses on variable selection wherein the outcome is related to imbalances in a few taxa. Given the difference in analytic philosophies, a significant community-level association supports the selected balance, while an insignificant community-level association does not truly invalidate the balance but prompts a warning, advising caution when interpreting the selected balance.

## 3 Illustrating examples

The first example is a study on graft-versus-host disease (GvHD) (Golob *et al.* 2017), a fatal complication of hematopoietic cell transplantation (HCT) and associated with the gut microbiome (Staffas *et al.* 2017, Andermann *et al.* 2018, Shono and van den Brink 2018, Fredricks 2019). Here, we aim to find gut microbial signatures measured right after HCT to describe the OS and time to the GvHD. The processed data contained 63 recipients, and the 16S rRNA microbiome data were aggregated to the genus level with rare taxa (relative abundance < 0.01%) removed. Details of the original and processed GvHD data are deferred to Section 3 of the Supplementary Information. The Cox proportional hazards model was used for analyzing OS and the parametric survival model assuming Weibull distribution was employed for time-to-GvHD. No covariates were adjusted and all other arguments were kept as the default options. The community-level association test showed that the gut microbiota is significantly associated with OS (omnibus *P*-value = .03) but not with time-to-GvHD (omnibus *P*-value = .82).

The global balance selected for OS by SurvBal includes six taxa increasing the risk of death $X_{+}^{\text{OS}}$={*Schaalia*, *Streptococcus*, *Eubacterium*, *Evtepia*, *Terrisporobacter*, *Ruminococcaceae*}, and nine taxa decreasing the risk $X_{-}^{\text{OS}}$={*Gemmiger*, *Phascolarctobacterium*, *Eggerthella*, *Agathobaculum*, *Christensenellaceae*, *Enterococcus*, *Pseudoflavonifractor*/*Clostridium*, *Collinsella*, *Romboutsia*}. Figure 1B presents the predicted OS curves. For recipients with a lower balance score (-0.37, the first quartile of the 63 recipients’ balances), which means that there are lower relative abundances of taxa in $X_{+}^{\text{OS}}$ than in $X_{-}^{\text{OS}}$, their life expectancy is significantly longer than those with a higher balance score (0.90, the third quartile of the 63 recipients’ balances). The balance identified for time-to-GvHD comprises four taxa prolonging the time to the disease $X_{+}^{\text{GvHD}}$={*Coprococcus*, *Monoglobus*, *Veillonella*, *Clostridiales Family XIII. Incertae Sedis*}, and six taxa shortening the time to the onset $X_{-}^{\text{GvHD}}$={*Flintibacter*, *Streptococcus*, *Negativibacillus*, *Flavonifractor*, *Neglecta*, *Intestinibacter*}, which is described by the predicted time-to-GvHD curves in Fig. 1C. Caution is recommended when interpreting the balance for time-to-GvHD due to the insignificant community-level association between the gut microbiota and time-to-GvHD. Nevertheless, the selected balance still provides useful insights. In particular, the two balances for GvHD and mortality share *Streptococcus*, which shortens the time-to-GvHD ($X_{-}^{\text{GvHD}}$) and increases the risk of death ($X_{+}^{\text{OS}}$). The positive association between *Streptococcus* and GvHD has been confirmed in previous HCT studies (Khan *et al.* 2021, Lin *et al.* 2021). We also note that with 63 patients only, some results obtained are contrary to existing literature. For example, *Enterococcus* is included in $X_{-}^{\text{OS}}$ but has been linked with increased risk of GvHD (Stein-Thoeringer *et al.* 2019). The results are expected to improve with the recruitment of more patients.

The second example is a study on bacteriuria after kidney transplant (Magruder *et al.* 2019, 2020). Bacteriuria is a common complication leading to significant morbidity, while modulating the gut microbiota could be a promising preventive intervention. Therefore, we would like to find the gut microbiome balance right after the surgery to predict the time to the onset of *Escherichia coli* and *Enterococcus* bacteriuria. The processed data contained 163 recipients, and the 16S rRNA microbiome data were aggregated to the genus level with common taxa (relative abundance > 1%) maintained. Details of the original and processed KTx data are deferred to Section 3 of the Supplementary Information. The Cox and parametric models were used for *E.coli* and *Enterococcus* bacteriuria, respectively. Gender is a crucial risk factor for bacteriuria thus was adjusted in the analysis. MiRKAT-S concluded with insignificant community-level associations between the gut microbiota and time-to-*E.coli* bacteriuria (omnibus *P*-value = .77) and time-to-*Enterococcus* bacteriuria (omnibus *P*-value = .51) while adjusting for gender.

The balance determined for *E.coli* bacteriuria consists of three genera increasing the risk $X_{+}^{E.\mathrm{coli}}$={*Bacteroides*, *Subdoligranulum*, *Holdemanella*}, and three genera decreasing the risk $X_{-}^{E.\mathrm{coli}}$={*Oscillibacter*, *Lachnoclostridium*, *Blautia*}. The predicted time-to-*E.coli* bacteriuria is longer for recipients with lower relative abundances of taxa in $X_{+}^{E.\mathrm{coli}}$ than in $X_{-}^{E.\mathrm{coli}}$, and the risk of *E.coli* bacteriuria is exaggerated within female recipients (Fig. 1D). The balance selected for *Enterococcus* bacteriuria includes one genus extending the time to the disease $X_{+}^{\mathrm{Enterococcus}}$={*Blautia*}, and five genera expediting the onset $X_{-}^{\mathrm{Enterococcus}}$= {*Erysipelatoclostridium*, *Lactobacillus*, *Anaerostipes*, *Enterococcus*, *Eubacterium*}. The predicted time-to-*Enterococcus* bacteriuria is systematically shortened with a lower balance score (Fig. 1E). Although caution is recommended when interpreting the selected balances, they still offer valuable insights. For example, SurvBal successfully identifies *Enterococcus* as a risk factor for promoting *Enterococcus* bacteriuria, which is consistent with prior work (Magruder *et al.* 2019). *Blautia* is selected for both preventing the onset of *Enterococcus* ($X_{+}^{\mathrm{Enterococcus}}$) and decreasing the risk of *E.coli* bacteriuria ($X_{-}^{E.\mathrm{coli}}$). It is a common commensal bacteria in the gut and is associated with the production of butyrate, a short-chain fatty acid (SCFA) (Maturana and Cárdenas 2021). Sorbara *et al.* (2019) has shown that SCFAs can inhibit the growth of *E. coli* via intra-cellular acidification, suggesting a competitive role of this bacteria against *E. coli* in the gut.

## 4 Conclusion

We have developed a software, SurvBal, to enable the selection of compositional balances in microbiome profiling studies with censored time-to-event outcomes. It allows the users to flexibly incorporate covariates and choose their preferred model type and other options. A community-level test, MiRKAT-S, is integrated into SurvBal to evaluate the overall association between the microbial profile and the survival outcome. It acts as a pre-selection inspection, endorsing the selected balance when a significant community-level association exists, and recommending caution when interpreting the selected balance if the community-level association is not significant. We believe that the selected global balance offers valuable insights, guiding further clinical investigations into the microbial markers, which can be modulated to improve the time-to-event outcomes.

There are various directions to extend SurvBal. One direction is to improve the greedy selection algorithm, though currently accompanied by a community-level test, to better accommodate the null case when no associations exist. Another limitation is that the current modeling cannot handle the competing nature of multiple causes for the same event. As SurvBal is designed with a flexible architecture, it opens avenues for further development to address the gaps, such as adding regularization and incorporating competing risk analysis.

## Supplementary Material

btae612_Supplementary_Data

## Data Availability

GvHD and KTx data (sequencing and de-identified clinical data) are available in BioProject with accession number PRJNA1052666 and in dbGaP with accession number phs001879.v1.p1, respectively.
