# Supplementary material for: SurvBal: compositional microbiome balances for survival outcomes
Source: Bioinformatics. 2024 Oct 15;40(10):btae612. doi: 10.1093/bioinformatics/btae612 (PMC11639162; doi:10.1093/bioinformatics/btae612)
Supplement: btae612_Supplementary_Data [file btae612_supplementary_data.pdf]

Supplementary information for "SurvBal: compositional microbiome balances for survival outcomes"

Ying Li, Teresa Lee, Kai Marin, Xing Hua, Sujatha Srinivasan,
David N. Fredricks, John R. Lee, Wodan Ling

1 Details of the software

1.1 Data processing

Microbiome data are frequently sparse, i.e., contain excessive zeros, as some taxa are observed in only a subset of the subjects. Since SurvBal operates on the log scale of relative abundances, zeros need to be imputed. Here, to process the raw counts of the microbiome, SurvBal provides two commonly used imputation techniques within the field – the geometric Bayesian multiplicative replacement (GBM) (Martín-Fernández et al., 2015) (default), which is used in the original "selbal" software, or adding a small pseudo-count (typically 0.5) to all counts. The imputed counts will then be converted to relative abundance and taken log transformation. Furthermore, since some taxa are observed in only a small handful of subjects, SurvBal allows users to provide a threshold such that taxa with prevalence below the threshold will be dropped (default=10%). This thresholding is particularly necessary when using GBM. Users can also process the microbial counts following their preferred routines before feeding the data into SurvBal, e.g., filtering out low-abundance taxa.

The global community-level association test, MiRKAT-S (Plantinga et al., 2017), is conducted in the pre-processing step. The microbiome data is encoded in ecologically informative distances – Bray-Curtis distance (based on microbiome relative abundances after filtering) and Jaccard distance (based on microbiome presence-absence status after filtering), and then kernel matrices are constructed from the distance matrices. Based on the two kernel matrices, MiRKAT-S, a kernel machine regression framework, tests a range of true forms of association, including the association of the survival outcome with the presence-absence status or the abundance of the microbial profile, while adjusting for covariates if applicable. It finally provides an omnibus p-value that combines the distances via residual permutation. If there is no significant community-level association (omnibus p-value > 0.05), SurvBal will issue a warning message, advising caution when interpreting the final selected balance.

1.2 Choosing a model

SurvBal offers two types of survival models – the Cox proportional hazards and parametric survival models. The former is a semi-parametric model that assumes the hazards (instantaneous probability of failure) are proportional across stratification of covariates, while the latter assumes the survival time follows a particular distribution such as the Weibull distribution (all the options in the R package "survival" can be specified, from exponential to log-logistic distributions). SurvBal allows both models to incorporate covariates such as crucial biomedical markers and demographic information.

A taxon that increases the hazard naturally correlates with a shortened survival time. Thus, for usual regression analysis with a fixed set of independent variables, the coefficients estimated by a parametric survival model will be the opposite sign of those by the Cox model. However, for variable selection, such as here, to select a balance of taxa, the group of taxa is not fixed but to be selected based on either the Cox

or parametric survival model. Since the Cox and parametric survival models are fundamentally different in all aspects – from target variables, assumptions, to estimation algorithms, it is rare for them to select exactly the same balance of taxa, but it is certain that if a taxon appears in the numerator of the selected balance by the Cox model, *very likely*, it will be added to the denominator of the balance by the parametric survival model.

The Cox model is the default when there is no clear evidence that the survival times follow a specific parametric distribution. This is because the Cox model is semi-parametric, which is more robust to the unknown distribution of the survival times. By design, SurvBal is a greedy method, aiming to identify a microbial balance that shows any kind of association with the survival outcomes. Thus, even when the proportional hazards assumption is violated, the Cox model-based SurvBal still works and selects a balance of taxa, only that interpreting the effect size is more challenging. In particular, the estimated hazard ratio reflects the average hazard ratio over the course of follow-up. This interpretation is less desirable but is still commonly done within modern studies of time-to-event outcomes, e.g., studies of checkpoint blockade in melanoma (Visconti et al., 2023; Tenorio-Pedraza et al., 2023; Qin et al., 2022). Nonetheless, careful assessment of assumptions is important. It is recommended to use functions such as *cox.zph* (from the R package "survival") to check if the final reported balance by the Cox model-based SurvBal meets the proportional hazards assumption, and if not, to consider alternative models when a standard interpretation of the effect size is preferred.

1.3 Selecting the global balance

1.3.1 Searching for the initial pair

First, SurvBal loops through all the possible pairs of the K taxa and determines the pair that is most correlated with the survival outcome. There are two combinations for each pair of taxa r and s. Via the Cox model, for example, $\log X_{ir} - \log X_{is}$ assumes that taxon r is positively associated with the hazard and taxon s is negatively associated with the hazard, while $\log X_{is} - \log X_{ir}$ assumes the opposite scenario. Thus, essentially, SurvBal does an exhaustive search, computing the Wald test p-values regarding the effects of the $\binom{K}{2}$ combinations (test $\gamma = 0$ in (2) of the manuscript), and picks the best pair with the minimal p-value while allocating the two bacteria into the numerator and denominator of the balance based on the sign of the pair's associated coefficient. Suppose $\log X_{ir} - \log X_{is}$ achieves the smallest p-value and its coefficient is positive, then the initial balance will be

$$B_i^{(1)} \propto \log X_{ir} - \log X_{is}.$$

1.3.2 Forward step-wise selection

Given $B_i^{(1)}$, SurvBal searches through all the remaining taxa for the next one to include in the balance. For each taxon t (where t is not equal to r or s), we assess both ways to add it – adding it to the numerator or the denominator of the balance,

$$B_i^{(2+)} \propto \frac{1}{2} \sum_{j \in \{r,t\}} \log X_{ij} - \log X_{is} = \frac{1}{2}(\log X_{ir} + \log X_{it}) - \log X_{is},$$

$$B_i^{(2-)} \propto \log X_{ir} - \frac{1}{2} \sum_{j \in \{s,t\}} \log X_{ij} = \log X_{ir} - \frac{1}{2}(\log X_{is} + \log X_{it}).$$

Again, we calculate the Wald test p-values regarding the effects of the two candidate balances, $B_i^{(2+)}$ and $B_i^{(2-)}$. The procedure is repeated for each remaining taxon in turn. Taxon t that gives the smallest p-value among the $(K-2) \times 2$ choices will be selected, and if the p-value corresponds to $B_i^{(2+)}$ then taxon t goes

into the numerator, while if it corresponds to $B_i^{(2-)}$ then taxon t goes into the denominator. We repeat the selection forward. Likewise, given $B_i^{(u-1)}$, SurvBal determines $B_i^{(u)}$ by picking one more taxon from the remaining pool that excludes taxa already in $B_i^{(u-1)}$ and adding it into the numerator or the denominator of the balance. The iteration terminates when it hits the stopping criteria – the p-value of the current balance is larger than a specified threshold (default=0.15). If a sparser model is preferred, on top of the stopping p-value, one can turn on sequential testing to ensure a conservative selection. It essentially tests whether the survival model fit is significantly improved with $B_i^{(u)}$ than $B_i^{(u-1)}$ (default significance level=0.25). If the contribution of $B_i^{(u)}$ is not significantly different from that of $B_i^{(u-1)}$, we stop adding more taxa.

1.3.3 Picking the optimal model

Although we stopped adding taxa, we need to figure out which model in the selection sequence is the best. This is because, unlike the additive models, the p-values of $B_i^{(1)}, B_i^{(2)}, \ldots$ may not monotonically decrease. Then, the balance with the smallest p-value is a natural choice for the final optimal model. This is the "minimum p-value" strategy. However, before reaching the smallest p-value, the forward step-wise selection usually reaches a plateau – incorporating additional taxa makes a minimal contribution to the balance. Thus, alternatively, we can pick the model such that the decrement of its p-value to the next model is less than a specified threshold (default=15%) for the first time along the sequence. This "minimum decrement of p-value" strategy can stop screening $B_i^{(1)}, B_i^{(2)}, \ldots$ earlier, and the final model's balance contains a subset of the taxa of that by the "minimum p-value" strategy, while the model fit could be similar. To achieve moderate model complexity and easier interpretations, "minimum decrement of p-value" is the default. However, when sequential testing is enabled to achieve a conservative selection, "minimum p-value" is recommended as a compromise.

1.4 Output and visualization

In the output, SurvBal reports the selected global balance, including the selection path, the names of bacterial taxa included in the numerator and the denominator of the balance, and the value of balance computed for each subject. It also presents the final survival model, the Cox proportional hazards or parametric survival model, with the selected balance and covariates specified by the users. Finally, it provides a visualization of the balance by examining predicted survival curves stratified by representative quantiles of the balance. SurvBal is flexible for users to explore the selected balance visually, allowing them to define interested quantile levels of the balance (default=1st quartile, 3rd quartile), and also the significance level (default=0.05) for confidence intervals of those stratified survival curves.

1.5 Practical considerations for implementation

As described above, the SurvBal software involves many arguments. Supp Table S1 lists the arguments, their explanations, and default values. To implement SurvBal, one needs to provide the survival outcome (*Surv_obj*) and microbiome data (*data*), and also the covariates (*covariates*), if applicable. For the remaining arguments, it is recommended to keep the default values unless there is a special need for analysis.

Next, the computation of SurvBal could be time-consuming if there are a large number of taxa. The computational complexity is $O(K^2)$, where K is the number of taxa after processing. Searching for the initial pair needs $\binom{K}{2}$ times of model fitting. For the worst case, the forward step-wise selection needs $K-2, K-3, \ldots, 1$ times of model fitting in each iteration, respectively. The time cost of other steps not involving model fitting is negligible. Thus, the total cost is $\binom{K}{2} + K - 2 + K - 3 + \cdots + 1 = O(K^2)$.

2 Evaluation on simulated data

We carried out simulation studies in a range of settings to assess the precision and recall of SurvBal in selecting the compositional microbiome balance for survival outcomes.

We simulated data based on MOMS-PI (Fettweis et al., 2019), a real vaginal microbiome dataset from the integrative Human Microbiome Project (int, 2019), available from the HMP2Data package (Stansfield et al., 2020). After aggregation to the genus level, the starting data contains 56 taxa from 270 individuals. Based on it, we mimicked ALDEx2 (Fernandes et al., 2014) to simulate taxonomic read counts. Specifically, for each individual i, we regarded the observed count vector as the parameters to generate relative abundances from a Dirichlet distribution, and then multiplied them by the individual's observed library size to obtain the simulated read counts, $\boldsymbol{X}_i = (X_{i1}, X_{i2}, \ldots, X_{i56})$. We also simulated two covariates for each individual, $\boldsymbol{Z}_i = (Z_{i1}, Z_{i2})$, from a standard normal and a Bernoulli (0.5) distribution, independently of the microbiome profiles. Next, we simulated exponentially distributed survival times under the proportional hazards assumption,

$$T_i = \frac{-\log(U_i)}{\lambda \exp\{\gamma B_i^{\mathrm{PH}}(\boldsymbol{X}_+, \boldsymbol{X}_-) + \boldsymbol{Z}_i^\top \boldsymbol{\beta}\}},$$

where γ is the true effect size of the balance, $\boldsymbol{\beta} = (1,1)^\top$ is the true effect size of the covariates, λ is the scale parameter, and $U_i \sim \mathrm{Uniform}(0,1)$. Censoring times were simulated independently as $C_i \sim \mathrm{Exp}(\mu)$, and λ and μ were selected to achieve approximately 2% to 22% censoring.

Two simulation settings were considered, varying whether the taxa that constituted $B_i^{\mathrm{PH}}(\boldsymbol{X}_+, \boldsymbol{X}_-)$ were correlated with the remaining taxa. This is a critical consideration because a consistent selection can be made if and only if irrelevant variables are not too correlated with the relevant variables (Irrepresentable Condition, Zhao and Yu (2006)). In the Uncorrelated Setting, 8 taxa were chosen to form the balance (4 in the numerator, I_+, and 4 in the denominator, I_-, and a pseudo-count 0.5 was added to all read counts to avoid the logarithm of zero problem),

$$B_i^{\mathrm{PH}}(\boldsymbol{X}_+, \boldsymbol{X}_-) = \sqrt{2} \log \frac{(\prod_{j \in I_+} X_{ij})^{1/4}}{(\prod_{j \in I_-} X_{ij})^{1/4}},$$

and the 8 relevant taxa had minimal correlation with the irrelevant 48 taxa (Supp Fig S1). In the Correlated Setting, again, 8 taxa were chosen to form the balance, but they were correlated with some of the irrelevant taxa (Supp Fig S2). Under the Correlated Setting, we should evaluate SurvBal based on both the 8-taxa balance and the actual balance, which also incorporated the taxa that were not chosen but correlated with the 8. Under either setting, we considered 2 effect sizes of the balance, $\gamma = 1$ (small effect size) and $\gamma = 2$ (large effect size), together with 3 sample sizes, $n = 135$ (halved the size of the starting data, randomly selected without replacement), $n = 270$ (the size of the starting data), and $n = 540$ (doubled the size of the starting data, randomly selected with replacement).

We repeated the simulation 1,000 times under each scenario and applied 3 settings of SurvBal – Cox model, Cox model with sequential testing, and parametric model. All the remaining arguments were set to be the default values of the software, except that SurvBal - Cox + seq used pseudo-count (adding 0.5) to impute zeros of the microbiome data, and used the "minimum p-value" strategy as suggested in Section 1.3.3. Precision ($\frac{\#\text{ selected relevant taxa}}{\#\text{ selected taxa}}$) and recall ($\frac{\#\text{ selected relevant taxa}}{\#\text{ relevant taxa}}$) in selecting taxa for both the numerator and denominator of the balance were calculated (4 metrics in total). We note that because a taxon that increases the hazard is naturally associated with a shortened survival time, the numerator selected by SurvBal - parametric corresponded to the denominator of $B_i^{\mathrm{PH}}(\boldsymbol{X}_+, \boldsymbol{X}_-)$ and its selected denominator corresponded to the numerator of $B_i^{\mathrm{PH}}(\boldsymbol{X}_+, \boldsymbol{X}_-)$. Stratified by the different scenarios, averages of the metrics over the 1,000 rounds were summarized by barplots.

Supp Fig S3 presents the performance of SurvBal under the Uncorrelated Setting with a small effect size ($\gamma = 1$) of the microbial balance. When $n = 270$, SurvBal - Cox achieved 0.75+ precision and

recall for the numerator and 0.80+ precision and recall for the denominator. Enabling sequential testing improved the precision to approximately 0.95 while reducing the recall to 0.55+ as a compromise. SurvBal - parametric yielded 0.85+ precision and recall. With the halved sample size, SurvBal demonstrated diminished precision and recall but still kept all the metrics around or above 0.75 except the recall of SurBal - Cox + seq, the most conservative option. With the doubled sample size, the performance of SurvBal - Cox and Cox + seq was further improved, while the precision of SurvBal - parametric was decreased, though its recall was boosted. This is expected as the data were simulated under the proportional hazards assumption – the increased sample size would improve the performance of the Cox model-based SurvBal but encourage the misspecified SurvBal - parametric to include irrelevant taxa. With the larger effect size ($\gamma = 2$, Supp Fig S4), comparisons across settings of SurvBal and sample sizes were almost the same. In addition, all the metrics of SurvBal - Cox and Cox + seq were enhanced. SurvBal - parametric's recall for all sample sizes and precision for $n = 135$ were improved, but precision for $n = 270, 540$ was diminished.

Under the Correlated Setting (top panels of Supp Figs S5 and S6), all settings of SurvBal demonstrated equivalent or even improved performance as compared to that under the Uncorrelated Setting, showing the robustness of SurvBal to the correlation structure of the bacterial taxa. Specifically, when $\gamma = 1$ and $n = 270$, almost all the metrics of SurvBal achieved 0.85+, only that the recall of the conservative SurvBal - Cox + seq and the precision of the misspecified SurvBal - parametric were around 0.60 - 0.80. Importantly, based on the actual balance that also incorporated the taxa that were not chosen but correlated with the relevant ones (bottom panels of Supp Figs S5 and S6), all the metrics were improved – most of them were boosted to 0.90+ or even close to 1.00 while the only exceptions were, again, the recall of SurvBal - Cox + seq and the precision of SurvBal - parametric. Same as that under the Uncorrelated Setting, the halved sample size reduced the precision and recall, the doubled sample size improved the performance except for the precision of SurvBal - parametric, and the larger effect size of the microbial balance ($\gamma = 2$) enhanced all the metrics except the precision of SurvBal - parametric.

In summary, the simulation studies proved the reliability of SurvBal. Under various scenarios, SurvBal consistently produced satisfactory precision and recall in selecting the balance of taxa for survival outcomes. It was robust to the correlation structure among the taxa, providing comparable performance under the Uncorrelated and Correlated Settings. Finally, SurvBal - Cox model showed stable and adequate precision and recall; enabling sequential testing ensured a conservative selection, which is recommended when the users focus more on the precision, while SurvBal - parametric is recommended when there is clear evidence that the survival times follow a specific parametric distribution, otherwise, though its recall is high its precision could be diminished.

3 Details of the illustrating examples

In this section, we provided more details about the two illustrating examples, GvHD and KTx.

Supp Fig S7 displays the Kaplan-Meier survival curves of the time to GvHD and the overall survival based on the GvHD data, with the corresponding tables of number of patients at risk. Supp Table S2 presents the summary statistics of the microbiome data before and after filtering. For the GvHD study, we would like to achieve initial screening without posing strict criteria on the taxa to be selected, so a general filtering threshold, relative abundance $< 0.01\%$, was used to omit rare taxa only.

Supp Fig S8 shows the stratified Kaplan-Meier survival curves of the time to *Escherichia coli* and *Enterococcus* bacteriuria according to gender based on the KTx data, with the corresponding tables of number of patients at risk. Supp Table S2 presents the summary statistics of the microbiome data before and after filtering. For the KTx study, the goal is to devise microbiota-based interventions. Thus, only common taxa that are easier to be modulated should be included, and the threshold of filtering was set to be relative abundance $> 1\%$. This is consistent with prior analyses on the KTx study (Lee et al., 2019b,a; Zhang et al., 2021).

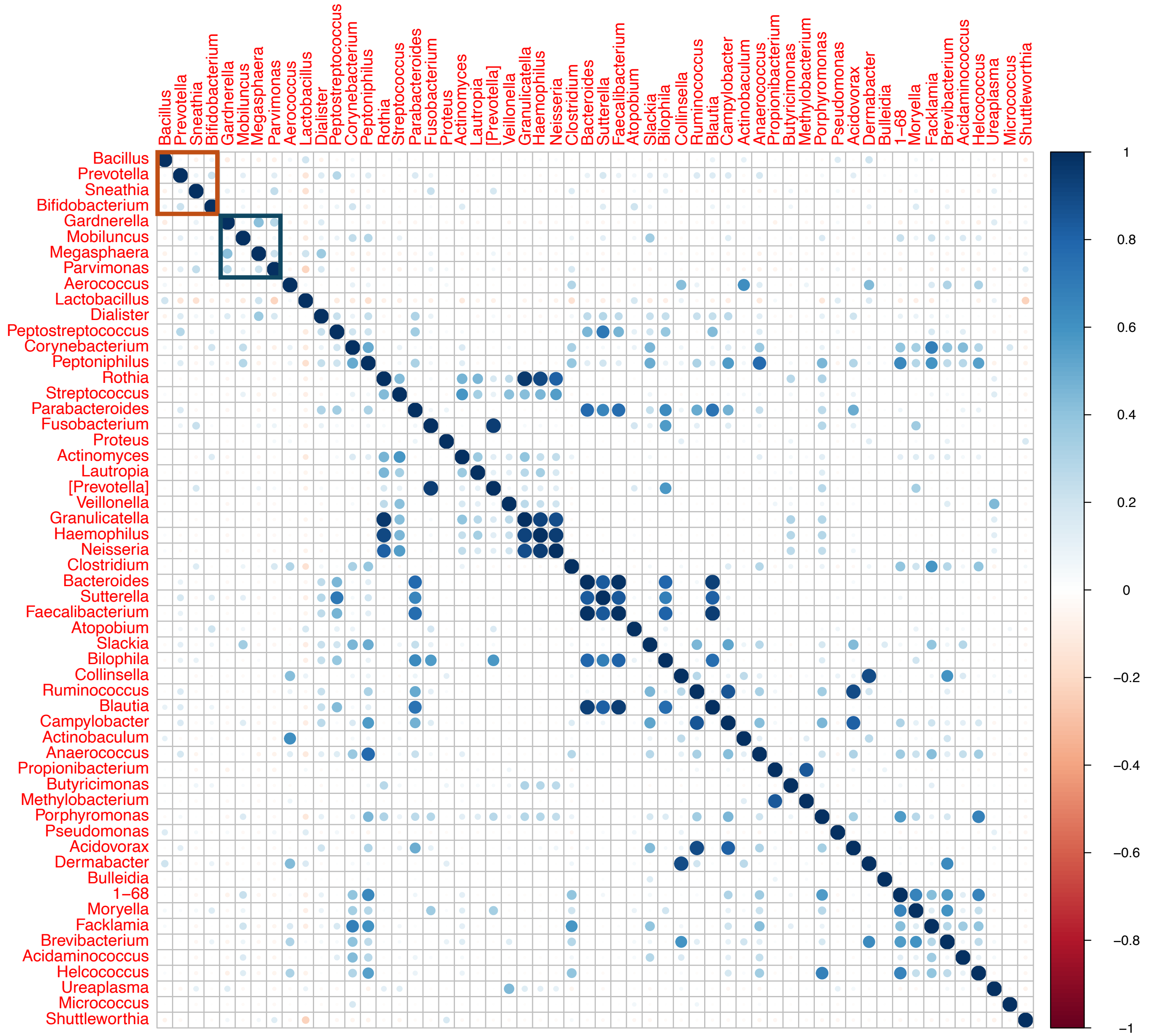

Figure S1: Uncorrelated Setting of simulation studies. Correlation heatmap of the 56 taxa from the original MOMS-PI data, with the red box highlighting the 4 taxa included in the numerator of $B_i^{\mathrm{PH}}(\boldsymbol{X}_+, \boldsymbol{X}_-)$, and the blue box highlighting the 4 taxa included in the denominator of $B_i^{\mathrm{PH}}(\boldsymbol{X}_+, \boldsymbol{X}_-)$. The 8 relevant taxa were chosen to have minimal correlation with the 48 irrelevant taxa.

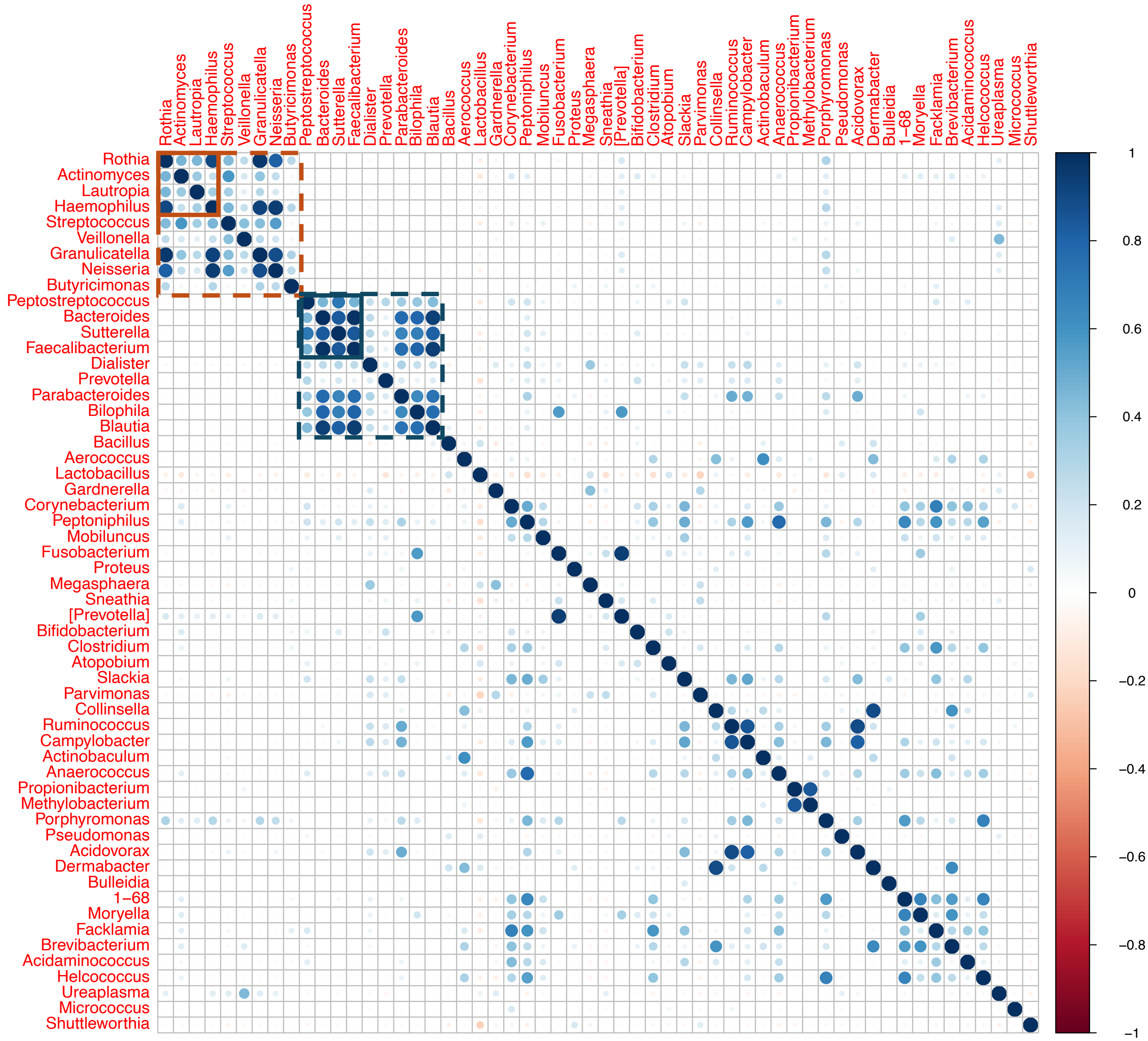

Figure S2: Correlated Setting of simulation studies. Correlation heatmap of the 56 taxa from the original MOMS-PI data, with the red solid box highlighting the 4 taxa included in the numerator of $B_i^{\mathrm{PH}}(\boldsymbol{X}_+, \boldsymbol{X}_-)$, and the blue solid box highlighting the 4 taxa included in the denominator of $B_i^{\mathrm{PH}}(\boldsymbol{X}_+, \boldsymbol{X}_-)$. The 8 relevant taxa were chosen to be correlated with some of the irrelevant taxa – the red dashed box includes the irrelevant taxa that were correlated with the relevant ones in the numerator, and the blue dashed box includes the irrelevant taxa that were correlated with the relevant ones in the denominator.

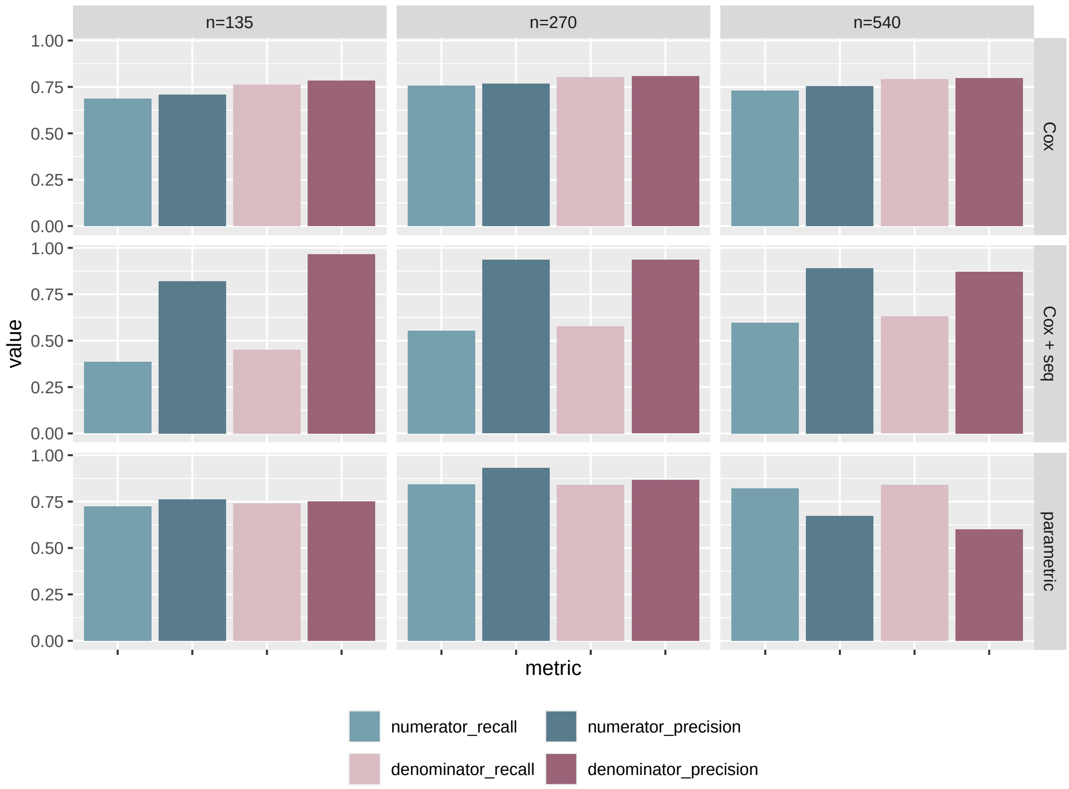

Figure S3: Simulation results under the Uncorrelated Setting with a small effect size of the compositional microbiome balance ($\gamma = 1$). 3 samples sizes – $n = 135, 270$, and 540, and 3 settings of SurvBal – Cox model, Cox model + sequential testing, and parametric model, were investigated. The averaged precision and recall in selecting the taxa for the numerator and denominator of $B_i^{\mathrm{PH}}(\boldsymbol{X}_+, \boldsymbol{X}_-)$ over 1,000 rounded were summarized in barplots.

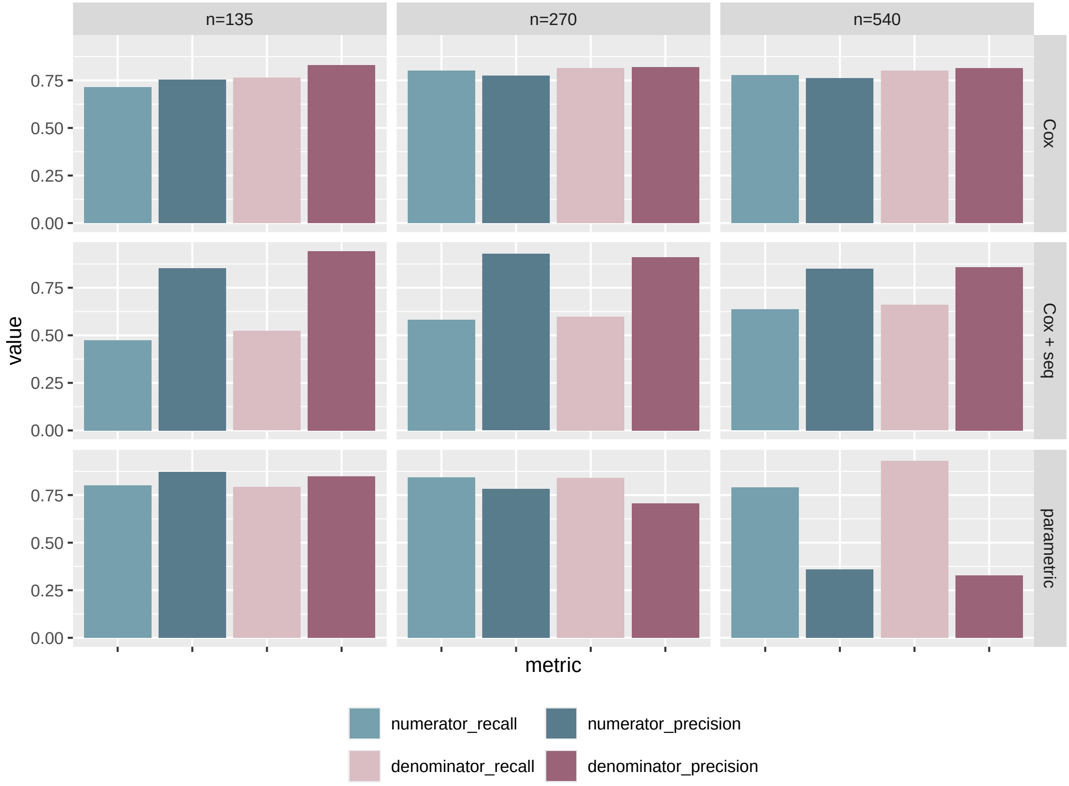

Figure S4: Simulation results under the Uncorrelated Setting with a large effect size of the compositional microbiome balance ($\gamma = 2$). 3 samples sizes – $n = 135, 270$, and 540, and 3 settings of SurvBal – Cox model, Cox model + sequential testing, and parametric model, were investigated. The averaged precision and recall in selecting the taxa for the numerator and denominator of $B_i^{\mathrm{PH}}(\boldsymbol{X}_+, \boldsymbol{X}_-)$ over 1,000 rounded were summarized in barplots.

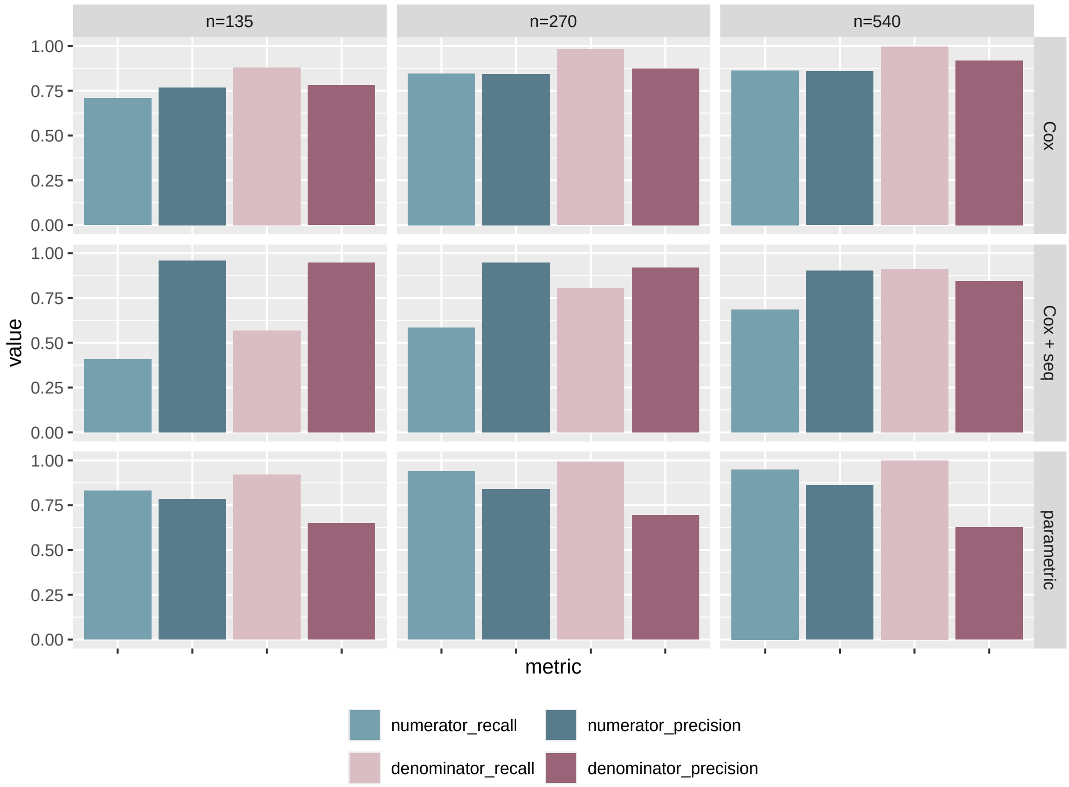

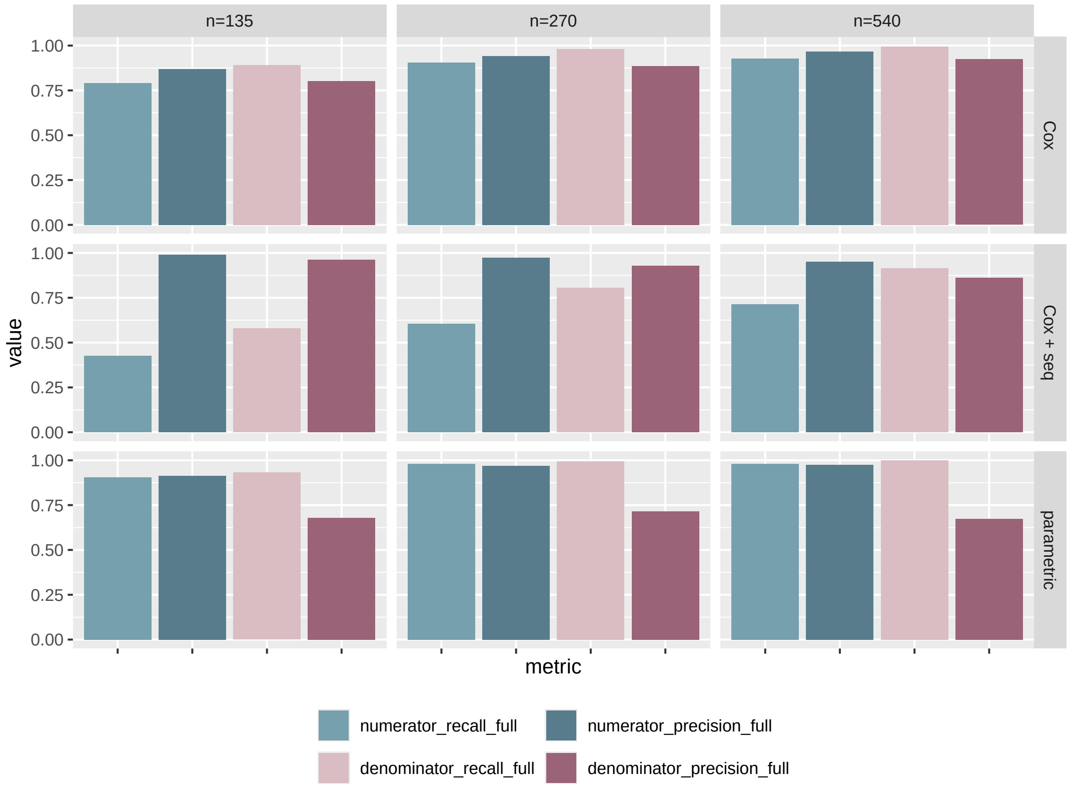

Figure S5: Simulation results under the Correlated Setting with a small effect size of the compositional microbiome balance ($\gamma = 1$). 3 samples sizes – $n = 135, 270$, and 540, and 3 settings of SurvBal – Cox model, Cox model + sequential testing, and parametric model, were investigated. The averaged precision and recall in selecting the taxa for the numerator and denominator of $B_i^{\text{PH}}(\boldsymbol{X}_+, \boldsymbol{X}_-)$ over 1,000 rounded were summarized in barplots. The evaluation was based on the 8-taxa balance (top panel) and also the actual balance that incorporated the taxa that were not chosen but correlated with the 8 (bottom panel).

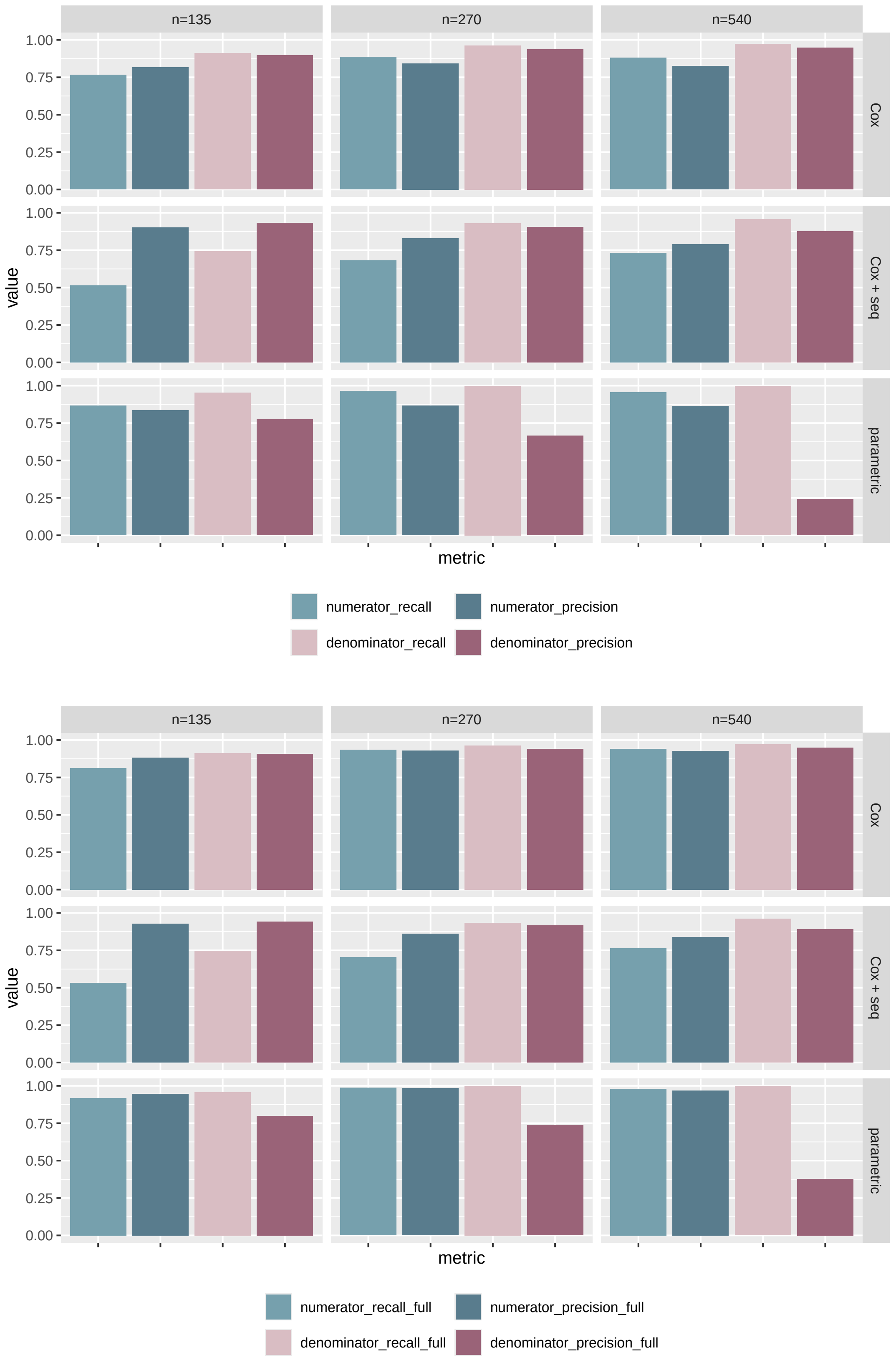

Figure S6: Simulation results under the Correlated Setting with a large effect size of the compositional microbiome balance ($\gamma = 2$). 3 samples sizes – $n = 135, 270,$ and 540, and 3 settings of SurvBal – Cox model, Cox model + sequential testing, and parametric model, were investigated. The averaged precision and recall in selecting the taxa for the numerator and denominator of $B_i^{\mathrm{PH}}(\boldsymbol{X}_+, \boldsymbol{X}_-)$ over 1,000 rounded were summarized in barplots. The evaluation was based on the 8-taxa balance (top panel) and also the actual balance that incorporated the taxa that were not chosen but correlated with the 8 (bottom panel).

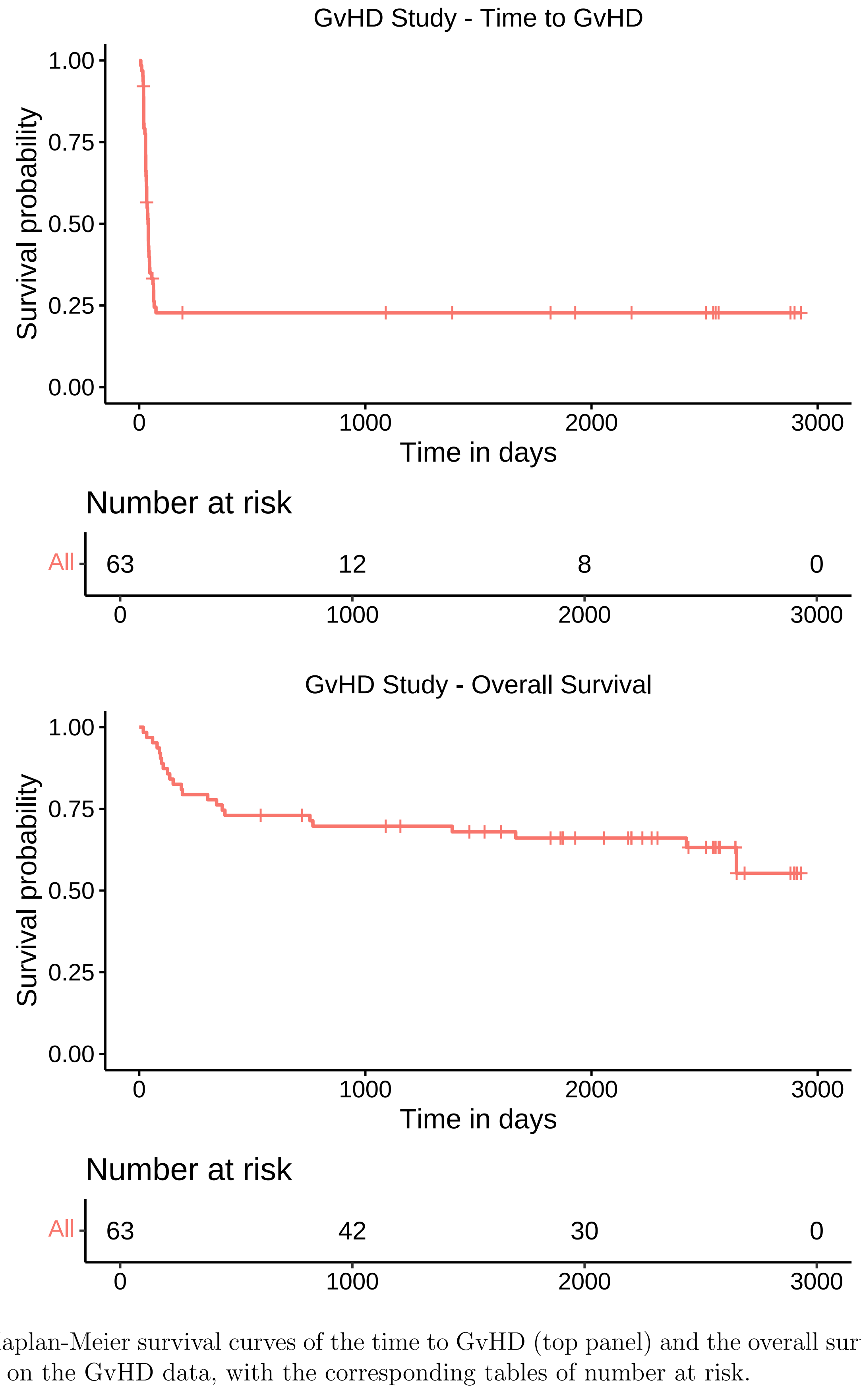

Figure S7: Kaplan-Meier survival curves of the time to GvHD (top panel) and the overall survival (bottom panel) based on the GvHD data, with the corresponding tables of number at risk.

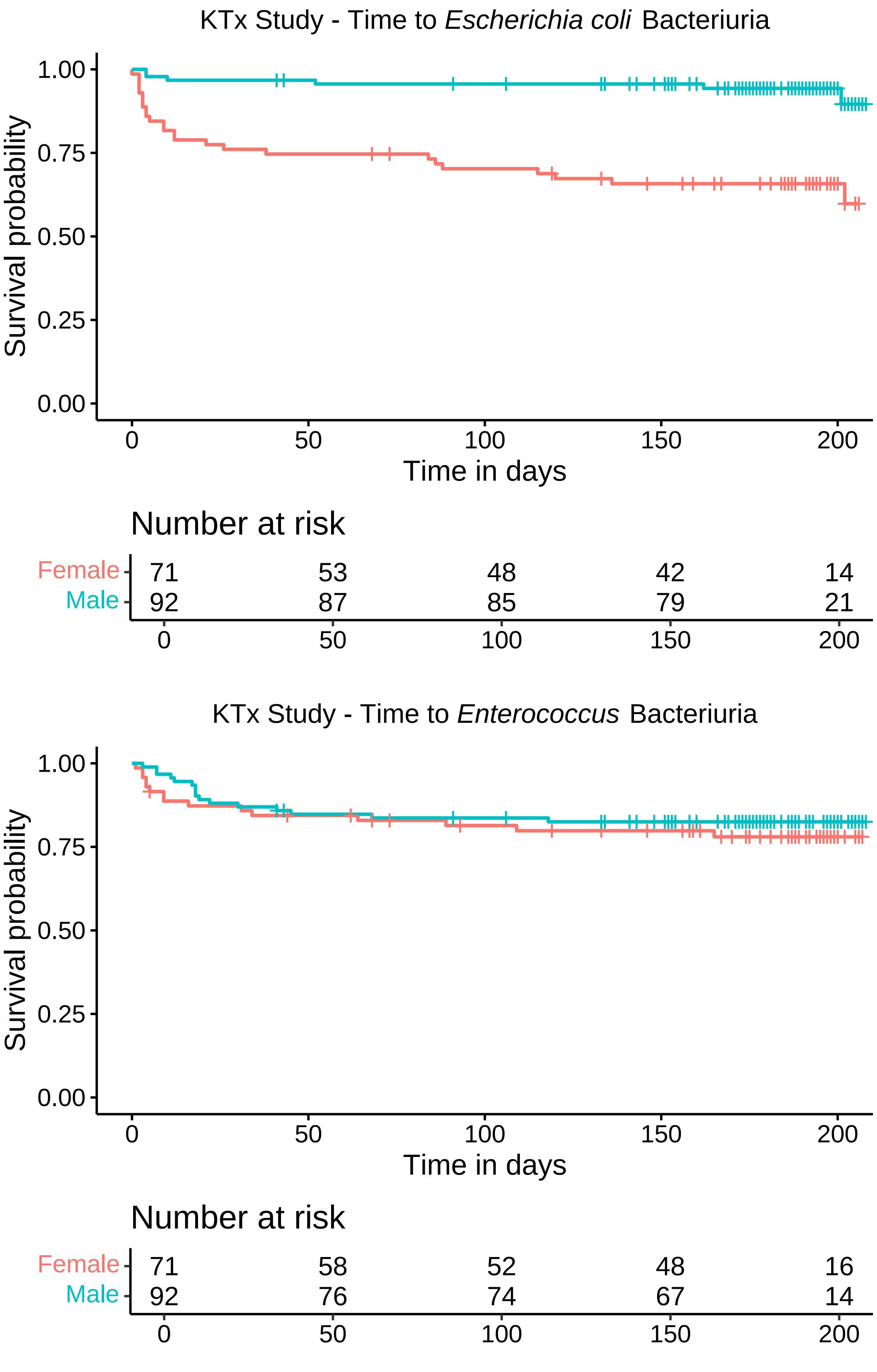

Figure S8: Stratified Kaplan-Meier survival curves of the time to *Escherichia coli* bacteriuria (top panel) and *Enterococcus* bacteriuria (bottom panel) according to gender based on the KTx data, with the corresponding tables of number at risk.

Table S1: Arguments of the SurvBal software with explanations and default values.

Argument	Explanation	Default
Surv_obj	An object of class Surv generated by R function Surv from R package survival	
data	The raw taxon count table, where rows are the samples, columns are the taxa	
covariates	A data frame containing important covariates for adjustment, which could be a mixture of continuous and discrete variables	NULL
min_prevalence	The minimum prevalence of taxa that could be considered in the selection procedure	0.1
mult_repl	Indicates which algorithm is used to process the raw taxon count table. If TRUE, the geometric Bayesian multiplicative replacement method is used to impute the inflated zeros. Otherwise, a small pseudo-count (0.5) will be added to all raw counts. Either processed data will be converted to log of relative abundance	TRUE
model	Specifies which kind of survival regression model is built. The options are "coxph" and "parametric"	coxph
dist	Specifies which kind of parametric distribution is used if model is "parametric". The options include "weibull", "exponential", "gaussian", "logistic", "lognormal" and "loglogistic"	weibull
stopping_pvalue	The threshold of p-value to stop the forward search. The forward search where, at each step, a new taxon is added to the existing balance, will be stopped if the resulting p-value is larger than stopping_pvalue	0.15
sequential_test	Indicates whether sequential testing is used to stop the forward search. If TRUE, the forward search where, at each step, a new taxon is added to the existing balance, will be stopped if the new balance is not significantly different from the current one in the survival regression model	FALSE
sequential_alpha	The level of significance for the sequential testing if sequential_test is TRUE	0.25
selection_criterion	The criterion to select the global balance in the final model. If "min_pvalue" is used, the balance with the smallest p-value along the forward selection path will be selected. If "min_decrement_pvalue" is used, the decrement of p-value along the forward selection path will be calculated, the balance before the first decrement that is smaller than selection_threshold will be selected	min_decrement_pvalue
selection_threshold	A threshold of p-value decrement used for balance selection in the final model if selection_criterion is "min_decrement_pvalue"	0.15
quantile_plotted	Specifies the quantiles of the final selected balance that will be shown in the survival plot	c(0.25, 0.75)
alpha	The level of significance for survival plot of the final selected balance. The corresponding confidence intervals will be shown in the survival plot	0.05

Table S2: Summary statistics for the microbiome data in the illustrating examples.

		GvHD	KTx
Before filtering	Number of taxa	193	245
	Average non-zero rate	0.1795	0.2852
	Average mean of relative abundance	0.0052	0.0041
	Average std of relative abundance	0.0121	0.0085
	Average CV of relative abundance	4.7621	6.2190
After filtering	Number of taxa	141	24
	Average non-zero rate	0.2358	0.8280
	Average mean of relative abundance	0.0071	0.0417
	Average std of relative abundance	0.0165	0.0699
	Average CV of relative abundance	3.9793	2.0803

References

The integrative human microbiome project. *Nature*, 569(7758):641–648, 2019.

A. D. Fernandes, J. N. Reid, J. M. Macklaim, T. A. McMurrough, D. R. Edgell, and G. B. Gloor. Unifying the analysis of high-throughput sequencing datasets: characterizing rna-seq, 16s rrna gene sequencing and selective growth experiments by compositional data analysis. *Microbiome*, 2:1–13, 2014.

J. M. Fettweis, M. G. Serrano, J. P. Brooks, D. J. Edwards, P. H. Girerd, H. I. Parikh, B. Huang, T. J. Arodz, L. Edupuganti, A. L. Glascock, et al. The vaginal microbiome and preterm birth. *Nature medicine*, 25(6):1012–1021, 2019.

J. R. Lee, J. Huang, M. Magruder, L. T. Zhang, C. Gong, A. N. Sholi, S. Albakry, E. Edusei, T. Muthukumar, M. Lubetzky, et al. Butyrate-producing gut bacteria and viral infections in kidney transplant recipients: a pilot study. *Transplant Infectious Disease*, 21(6):e13180, 2019a.

J. R. Lee, M. Magruder, L. Zhang, L. F. Westblade, M. J. Satlin, A. Robertson, E. Edusei, C. Crawford, L. Ling, Y. Taur, et al. Gut microbiota dysbiosis and diarrhea in kidney transplant recipients. *American Journal of Transplantation*, 19(2):488–500, 2019b.

J.-A. Martín-Fernández, K. Hron, M. Templ, P. Filzmoser, and J. Palarea-Albaladejo. Bayesian-multiplicative treatment of count zeros in compositional data sets. *Statistical Modelling*, 15(2):134–158, 2015.

A. Plantinga, X. Zhan, N. Zhao, J. Chen, R. R. Jenq, and M. C. Wu. Mirkat-s: a community-level test of association between the microbiota and survival times. *Microbiome*, 5:1–13, 2017.

Q. Qin, T. Jun, B. Wang, V. G. Patel, G. Mellgard, X. Zhong, M. Gogerly-Moragoda, A. B. Parikh, A. Leiter, E. J. Gallagher, et al. Clinical factors associated with outcome in solid tumor patients treated with immune-checkpoint inhibitors: a single institution retrospective analysis. *Discover Oncology*, 13 (1):73, 2022.

J. Stansfield, E. Smirnova, N. Zhao, J. Fettweis, L. Waldron, and M. Dozmorov. Hmp2data: 16s rrna sequencing data from the human microbiome project 2. *R package version*, 1(0), 2020.

J. M. Tenorio-Pedraza, J. Lippert, R. Burghaus, and C. Scheerans. Meta-analysis of preclinical measures of efficacy in immune checkpoint blockade therapies and comparison to clinical efficacy estimates. *Translational Medicine Communications*, 8(1):17, 2023.

A. Visconti, N. Rossi, H. Deriš, K. A. Lee, M. Hanić, I. Trbojević-Akmačić, A. M. Thomas, L. A. Bolte, J. R. Björk, J. S. Hooiveld-Noeken, et al. Total serum n-glycans associate with response to immune checkpoint inhibition therapy and survival in patients with advanced melanoma. *Bmc cancer*, 23(1):166, 2023.

L. T. Zhang, L. F. Westblade, F. Iqbal, M. R. Taylor, A. Chung, M. J. Satlin, M. Magruder, E. Edusei, S. Albakry, B. Botticelli, et al. Gut microbiota profiles and fecal beta-glucuronidase activity in kidney transplant recipients with and without post-transplant diarrhea. *Clinical Transplantation*, 35(5):e14260, 2021.

P. Zhao and B. Yu. On model selection consistency of lasso. *The Journal of Machine Learning Research*, 7:2541–2563, 2006.
